# Comparing the clinical profile of non obese children with sleep apnea and snoring

**DOI:** 10.5935/1808-8694.20120004

**Published:** 2015-11-20

**Authors:** Daniele de Oliveira Soares Stefanini, Emília Leite de Barros, Renato Stefanini, Márcia Lurdes de Cássia Pradella-Hallinan, Shirley Shizue Nagata Pignatari, Reginaldo Raimundo Fujita

**Affiliations:** a MSc at UNIFESP (MD, Otorhinolaryngologist); b MD, Otorhinolaryngologist (MD, Otorhinolaryngologist); c MSc at UNIFESP (MD, Otorhinolaryngologist); d PhD at UNIFESP (Assisting Physician at UNIFESP); e Post-Doctoral Degree at UNIFESP (Adjunct Professor at UNIFESP); f PhD at UNIFESP (Professor adjunto na UNIFESP). UNIFESP

**Keywords:** child, metabolic diseases, polysomnography, sleep apnea, obstructive

## Abstract

Few studies in the literature have looked into the cardiovascular and metabolic effects of Obstructive Sleep Apnea Syndrome (OSAS) in children.

**Objective:**

This study aims to evaluate the metabolic profile of non-obese children with OSAS.

**Methods:**

Fifty-two children were enrolled in this study, 21 girls and 31 boys. Patients were divided into two groups: OSAS (28 children) and Snore (22 children) according to polysomnographic evaluation. All children were submitted to ENT examination, measurements of weight, height and blood pressure. Blood samples were tested for hemoglobin, hematocrit, fasting glucose, fasting insulin, triglycerides, total cholesterol, HDL, LDL, VLDL, TSH and T4. The gathered data sets were compared between groups and also within the OSAS group according to the severity of the syndrome.

**Results:**

The children from both groups had no alterations in blood pressure levels. The results of the blood tests were normal for both groups. Results of hemoglobin, hematocrit, and HDL were all significantly higher in the Snore group when compared to the OSAS group; by their turn, VLDL levels were higher in the OSAS group. There was no statistical difference between the groups based on OSAS severity.

**Conclusion:**

Non-obese children with OSAS present no significant alterations in metabolic tests or blood pressure levels.

## INTRODUCTION

Obstructive sleep apnea syndrome (OSAS) is a respiratory sleep disorder that affects adults and children. It is characterized by repeated episodes of upper airway obstruction accompanied by intermittent hypoxia and hypercapnia^1^.

Prevalence rates reported in the literature range between 0.7% and 3%. Incidence rates peak among preschoolers, as this age group is more affected by adenotonsillar hypertrophy, the main cause of OSAS in children^1^. The treatment of pediatric OSAS is adenotonsillectomy or, in rare cases, CPAP^1^, ^2^, ^3^.

OSAS may have severe clinical consequences such as cor pulmonale, in addition to other cardiovascular and metabolic disorders, delays in weight and height gain, facial and chest skeletal alterations, nocturnal enuresis, behavior and learning disorders, and cognitive impairments^1^^,^^4^.

The association between OSAS and systemic high blood pressure and other cardiovascular and metabolic diseases has been well documented^1^^,^^5^, ^6^, ^7^, ^8^. Sleep disorders and intermittent nocturnal hypoxemia in combination with OSAS have been related to metabolic disorders including altered glucose metabolism and dyslipidemia. Evidence suggests an association between OSAS and diabetes mellitus type 2, glucose intolerance, and insulin resistance^9^.

Studies in pediatric populations have shown an association between OSAS and alterations in systemic blood pressure levels^10^^,^^11^, echocardiographic findings^12^^,^^13^ and insulin resistance^14^^,^^15^. These alterations seem to be present mainly in children with moderate to severe OSAS and in obese kids^16^. Only a few studies have looked into the consequences of OSAS in non-obese children.

This study compared the clinical profiles of non-obese children with OSAS and primary snoring.

## METHOD

The study was approved by the Research Ethics Committee of the institution and granted permit CEP 1814/08. Pediatric patients seen in an outpatient setting between March of 2009 and August of 2010 were enrolled. The subjects were aged between three and 13 years and were predominantly mouth breathers. They had been snoring three or more days per week for at least six months and had a body mass index (BMI) equal to or under the 95th percentile based on age and gender. Obese and diabetic subjects and individuals with cardiovascular, metabolic or neuromuscular diseases, genetic syndromes or craniofacial malformations were excluded.

The patients underwent complete ENT examination and had their weight, height, and blood pressure (BP) measured. BP was measured with the patients seated after resting for at least 20 minutes. A proper cuff for the patients' age and weight was used. BP measurements were categorized as 'altered' or 'unaltered' in accordance with the second release of the table published by the *National Task Force on Hypertension of the National Heart, Lung and Blood Institute* endorsed by the American Academy of Pediatrics in 1987 and reviewed in 1996^17^^,^^18^. Systolic or diastolic BP values under the 90th percentile were considered normal.

Tonsillar hypertrophy was categorized in four degrees as per the scheme proposed by Brodsky^19^, while pharyngeal tonsil hypertrophy was assessed through nasopharyngeal endoscopic examination. Grades III and IV were considered as obstructive palatine tonsil hypertrophy. Obstruction caused by the pharyngeal tonsil was characterized when it occupied 70% or more of the cavum area.

All subjects underwent polysomnography. Obstructive apnea was characterized as the presence of inspiratory effort in the absence of airflow for at least two respiratory events. Hypopnea was defined as a reduction of at least 50% in airflow amplitude in the presence of inspiratory effort and oxyhemoglobin desaturation of at least 4%, or the subject awakening. The apnea hypopnea index (AHI) was defined as the set of obstructive and mixed (central and obstructive components) apnea and hypopnea episodes per hour of sleep^20^. OSAS was defined as the presence of AHI ≥ 1. If AHI ≥ 1 and < 5, the patient had mild OSAS; if AHI ≥ 5 and < 10, the subject had moderate OSAS; severe cases had AHI > 10.

Fasting blood samples were taken to assess hemoglobin (Hb), hematocrit (Ht), fasting glucose, fasting insulin, triglycerides, total cholesterol and cholesterol fractions (HDL, LDL, VLDL), TSH and free T4 levels.

Non-parametric tests were used in this study.

The test for equality of two proportions was used to compare whether the proportions of responses of two variables and/or their levels are statistically significant. The Mann-Whitney test was used to compare variables from independent samples two by two. The Kruskal-Wallis test was used to compare the three degrees of OSAS severity to all quantitative variables.

Spearman's rank correlation ratio was used to measure the correlation between all variables and the OSAS degree of severity. The correlation test was used to validate the correlations.

Significance was set at 0.05 (5%). All confidence intervals used in this study had a 95% statistical confidence level.

## RESULTS

Fifty-two children were enrolled in the study, 21 (40.38%) of them being girls and 31 (59.61%) boys. The patients were divided into two groups: OSAS (AHI ≥ 1 event per hour) and Snoring (AHI < 1 event per hour). Both groups were homogeneous in terms of gender and age. Twenty-eight children were included in the Snoring

Group (13 females and 15 males), while the OSAS Group had 24 subjects (8 females and 16 males). In the OSAS Group, eleven children had mild OSAS, seven had moderate OSAS, and six had severe OSAS. Mean ages were 8.07 years in the Snoring Group and 6.67 years in the OSAS Group.

All subjects had obstructive OSAS caused by hypertrophied lymphoid tissue. In the OSAS Group, 18 (75%) children had hypertrophied pharyngeal tonsils and 15 (62.5%) had hypertrophied tonsils. In the Snoring Group, 13 (46.4%) individuals had hypertrophied pharyngeal tonsils and 10 (35.7%) had hypertrophied tonsils.

The subjects from both groups had unaltered BP levels.

The groups were compared for quantitative variables age, BMI, Hb, Ht, fasting glucose, total cholesterol, cholesterol fractions, triglycerides, insulin, TSH, an free T4 as described on Table 1.

**Table 1:** Analysis of quantitative variables in Snoring and OSAS Groups.

	Snoring	OSAS	*p*
Age (years)	8.07 ± 2.75	6.67 ± 3.48	0.084
BMI (Kg/cm^2^)	16.91 ± 3.23	17.23 ± 4.37	0.653
Hb (g/dL)	13.41 ± 0.88	12.69 ± 0.80	0.004
Ht (%)	39.78 ± 2.24	38.15 ± 2.59	0.024
Glucose (mg/dL)	87.04 ± 6.17	85.29 ± 7.93	0.382
Cholesterol (mg/dL)	147.07 ± 22.62	149.38 ± 25.36	0.666
HDL (mg/dL)	49.39 ± 6.05	44.79 ± 7.05	0.009
LDL (mg/dL)	84.85 ± 20.38	86.76 ± 20.60	0.707
VLDL (mg/dL)	12.65 ± 4.71	17.83 ± 12.81	0.049
Triglycerides (mg/dL)	65.04 ± 23.68	89.17 ± 64.13	0.097
Insulin (μUI/mL)	3.48 ± 2.34	2.84 ± 2.41	0.142
TSH (μUI/mL)	2.16 ± 1.17	2.29 ± 0.84	0.409
Free T4 (ng/dL)	0.94 ± 0.11	1.33 ± 1.79	0.308

OSAS: obstructive sleep apnea syndrome; n: number of subjects; BMI: body mass index; Hb: Hemoglobin; Ht: Hematocrit; HDL: high density lipoprotein; LDL: low density lipoprotein; VLDL: very low density lipoprotein; TSH: thyroid-stimulating hormone; free T4: thyroxine.

The studied variables had normal values in both groups. However, statistically significant differences were found between the groups for Hb, Ht, HDL, and VLDL. Among these, only VLDL was higher in the OSAS Group than in the Snoring Group.

The subjects in the OSAS Group were analyzed to compare the three degrees of syndrome severity and their quantitative variables. The differences observed between the subjects with various degrees of OSAS severity were not statistically significant.

The possible correlations between all variables and OSAS severity were analyzed for the OSAS Group (Table 2).

**Table 2:** Correlation between variables and OSAS severity

	Correlation	*p*-value
Age (years)	3.60%	0.866
Hb (g/dL)	13.20%	0.538
Ht (%)	2.10%	0.922
Glucose (mg/dL)	-36.50%	0.079
Cholesterol (mg/dL)	-4.20%	0.844
HDL (mg/dL)	7.80%	0.717
LDL (mg/dL)	-2.60%	0.904
VLDL (mg/dL)	4.80%	0.822
Triglycerides (mg/dL)	4.80%	0.822
Insulin (μUI/mL)	-46.60%	0.022
TSH (μUI/mL)	-11.80%	0.582
Free T4 (ng/dL)	40.50%	0.049

OSAS: obstructive sleep apnea syndrome; Hb: Hemoglobin; Ht: Hematocrit; HDL: high density lipoprotein; LDL: low density lipoprotein; VLDL: very low density lipoprotein; TSH: thyroid-stimulating hormone; free T4: thyroxine.

There was a negative correlation between the degree of severity and insulin (-46.6%) and a positive one with free T4 (40,5%). Both correlations were rated as Fair.

## DISCUSSION

Many papers in the literature have shown that children with OSAS have altered BP levels^10^^,^^21^, ^22^. Altered BP levels were not observed in this study for the non-obese children of either of the groups. Kaditis et al.^16^ also failed to show altered BP levels in non-obese children with OSAS, but we found significantly higher BP values in the OSAS Group when compared to subjects in the control group. The study done by Amin et al.^12^ in 2002 did not reveal altered BP levels in children with OSAS or in primary snorers.

The metabolic indices of the patients in both groups were not altered. Only VLDL was increased and HDL cholesterol was significantly lower in children with OSAS. Hemoglobin and hematocrit levels were significantly higher in the patients without OSAS. Kaditis et al.^16^ studied non-obese children with OSAS and did not find differences in total cholesterol, cholesterol fractions, triglycerides, and insulin levels when compared to children without OSAS.

Only patients with OSAS were checked to see whether there was any variation in their metabolic indices in association with OSAS severity, but no statistically significant differences were found between OSAS severity and the analyzed metabolic indices. It was observed that increasing degrees of disease severity led to lower insulin levels and higher free T4 values.

These results vary from the ones reported by de la Eva et al.^14^, in which a positive correlation was established between OSAS severity and insulin levels in children regardless of BMI. In China, obese children with OSAS were studied, and increased levels of insulin were found in this group, in a positive correlation with OSAS severity^23^. Redline et al.^10^ studied a group of teens and showed that adolescents with sleep respiratory disorders have seven times more chance of having metabolic alterations than adolescents with no sleep disorders. After adjustments for gender and BMI, these adolescents had higher levels of BP, insulin, and LDL cholesterol.

Nonetheless, the study conducted by Tauman et al.^24^ verified that obesity, and not OSAS, is the determining factor for insulin resistance and metabolic alterations seen in their cohorts. This study can explain why our study and in the one by Kaditis no metabolic alterations were found in non-obese children with and without OSAS. It appears that fat tissue plays an important role as a mediator for cytokines, growth factors, and sex steroids, which alter the action and secretion of insulin^25^.

A longitudinal study done with children showed that metabolic alterations are more evident between 10 and 19 years of age^26^. The differing results seen in the studies mentioned above may be related to the variations in age of the studied populations. Positive findings may prevail in older children and in adolescents, as they have been enduring the consequences of OSAS for longer than younger subjects, not to mention the impact of puberal changes.

Another confounding factor in OSAS pediatric studies is obesity, which in some papers is reported as a determining factor for the presence of metabolic alterations while not in others. More studies are required to look into children with OSAS and metabolic alterations by separating obese from non-obese subjects and analyzing them by age range.

Our study looked only at non-obese children to isolate the consequences associated with OSAS from obesity. Age is known to affect the onset of metabolic alterations. Therefore, it is important to divide subjects based on their ages. However, given the difficulties inherent to polysomnographic examination, our sample had a limited number of subjects and could not be further divided into age-based subgroups.

The literature on adult OSAS is extensive, but few studies have been carried out on pediatric sleep respiratory disorders. The papers on children with OSAS and metabolic and inflammatory disorders have diverging results, mainly because of the myriad of methods employed to categorize OSAS and the variations in the data related to subject age. Adolescents appear to have different metabolic characteristics than younger children, but they have been studied together in most papers, which have thus failed to recognize the age-related variations.

## CONCLUSION

Non-obese children with OSAS did not present significant blood pressure or metabolic alterations when compared to pediatric primary snoring patients.
